# Convergent Sensing: Integrating Biometric and Environmental Monitoring in Next-Generation Wearables

**DOI:** 10.3390/bios16010043

**Published:** 2026-01-04

**Authors:** Maria Guarnaccia, Antonio Gianmaria Spampinato, Enrico Alessi, Sebastiano Cavallaro

**Affiliations:** 1 Institute for Biomedical Research and Innovation, National Research Council, 95126 Catania, Italy; maria.guarnaccia@cnr.it; 2 Xenia—Software Solution, Aci Castello, 95021 Catania, Italy; aspampinato@xeniaprogetti.it; 3 Analog, Power & Discretes, MEMS and Sensors Group, Central R & D, STMicroelectronics, 95121 Catania, Italy; enrico.alessi@st.com

**Keywords:** wearable biosensors, multi-modal sensing, biometric monitoring, environmental sensing, data fusion, digital health, galvanic skin response (GSR)

## Abstract

The convergence of biometric and environmental sensing represents a transformative advancement in wearable technology, moving beyond single-parameter tracking towards a holistic, context-aware paradigm for health monitoring. This review comprehensively examines the landscape of multi-modal wearable devices that simultaneously capture physiological data, such as electrodermal activity (EDA), electrocardiogram (ECG), heart rate variability (HRV), and body temperature, alongside environmental exposures, including air quality, ambient temperature, and atmospheric pressure. We analyze the fundamental sensing technologies, data fusion methodologies, and the critical importance of contextualizing physiological signals within an individual’s environment to disambiguate health states. A detailed survey of existing commercial and research-grade devices highlights a growing, yet still limited, integration of these domains. As a central case study, we present an integrated prototype, which exemplifies this approach by fusing data from inertial, environmental, and physiological sensors to generate intuitive, composite indices for stress, fitness, and comfort, visualized via a polar graph. Finally, we discuss the significant challenges and future directions for this field, including clinical validation, data security, and power management, underscoring the potential of convergent sensing to revolutionize personalized, predictive healthcare.

## 1. Introduction

**Figure 1:**
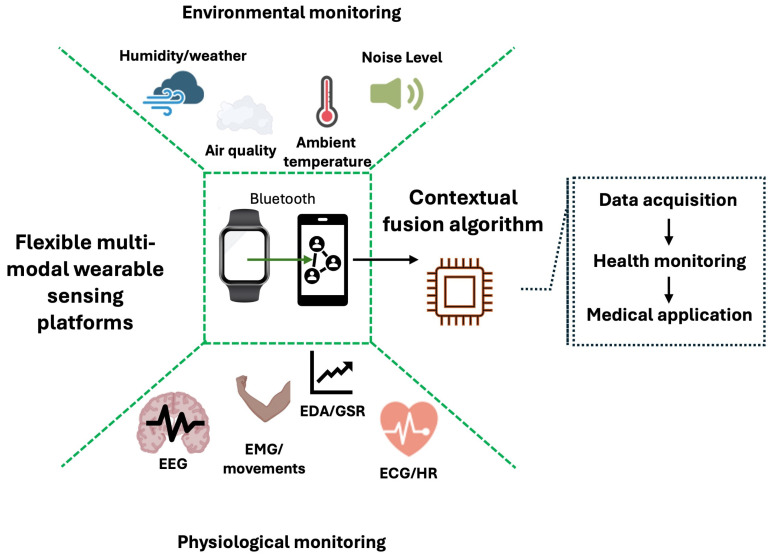
Contextual framework for biometric–environmental integration.

The landscape of wearable health monitoring has evolved dramatically from basic activity tracking to sophisticated multi-parameter systems capable of capturing comprehensive physiological and environmental data [1]. Integrated with AI, these wearables provide real-time, non-invasive, and continuous health tracking, becoming increasingly accessible and advanced [2]. This technological evolution represents a paradigm shift in personal health assessment, enabling unprecedented context-aware interpretation of biometric signals through the integration of internal physiological monitoring with external environmental and activity-based sensing [3,4]. Traditional wearable devices have primarily focused on isolated physiological parameters, critically lacking the environmental and activity context essential for accurate signal interpretation and clinically meaningful health insights [4]. The fundamental rationale for integrating biometric, environmental and activity sensing addresses a core limitation in conventional health monitoring approaches: human physiological responses are intrinsically modulated by both environmental conditions and physical activity level [5]. As illustrated in Figure 1, ambiguous biometric signals can only be properly interpreted when contextualized with complementary environmental and activity data. Ambient temperature directly influences thermoregulatory processes, air quality triggers inflammatory and stress responses, atmospheric pressure variations affect cardiovascular function, and physical activity levels directly impact metabolic and cardiovascular parameters [6]. Without this essential contextual layer, biometric data remain incomplete, potentially misleading, and of limited clinical utility. The conceptual framework presented in Figure 1 demonstrates how convergent sensing transforms ambiguous physiological readings into informed health assessments. This integrated approach enables precise differentiation between physiologically similar but etiologically distinct states, such as exercise-induced tachycardia versus stress-related palpitations, or environmentally triggered respiratory distress versus infectious respiratory conditions [7,8]. By providing this critical contextual layer, multi-modal wearables overcome the interpretative limitations that have constrained traditional single-domain monitoring systems [9]. This comprehensive review examines the technological foundations, implementation challenges, and clinical applications of convergent sensing platforms. We systematically analyze the complete spectrum of available biometric, environmental, and activity sensing modalities, survey the current landscape of multi-sensor wearable devices, and present a detailed case study of an advanced prototype that demonstrates the practical utility of this integrated approach. The convergence of these sensing domains enables truly personalized health monitoring that accounts for individual physiological responses within specific environmental and activity contexts, thereby paving the way for genuinely predictive and preventive healthcare interventions [10].

## 2. Fundamental Sensing Technologies

Modern wearable platforms incorporate increasingly diverse physiological sensing technologies that provide complementary insights into health status across three primary domains: biometric sensing, environmental sensing, and activity monitoring [9,11]. The strategic combination of these modalities creates a comprehensive health profile that far exceeds the diagnostic capabilities of any single sensor type, enabling more robust health assessment through cross-validation and data fusion [12].

### 2.1. Biometric Sensing Modalities

Biometric sensing captures the body’s physiological responses, which are influenced by both environmental conditions and physical activity levels.

Electrophysiological monitoring encompasses several sophisticated technologies for measuring the electrical signals generated by various physiological processes [13,14]. Electrocardiography (ECG) systems capture cardiac electrical activity through cutaneous electrodes, enabling basic heart rate monitoring, arrhythmia detection, HRV analysis, and myocardial ischemia assessment [15,16]. Electrodermal Activity (EDA) sensors measure skin conductance variations resulting from sweat gland activity controlled by the sympathetic nervous system, serving as highly sensitive indicators of autonomic arousal, emotional states, and cognitive load. Modern EDA systems incorporate sophisticated decomposition algorithms that separate signals into tonic (slow-changing) and phasic (fast-changing) components, providing insights into both background arousal levels and acute stress responses [17]. Electroencephalography (EEG) systems monitor electrical brain activity through scalp-mounted electrodes, offering insights into sleep architecture, cognitive states, and neurological disorders [18,19]. Electromyography (EMG) sensors detect skeletal muscle electrical activity, enabling assessment of muscle fatigue, movement intention, and neuromuscular disorders [20,21]. Sensory evoked potentials, including somatosensory evoked potential (SSEP), motor evoked potential (MEP), brainstem auditory evoked potential (BAEP), and visual evoked potential (VEP) measure the nervous system’s response to sensory or motor stimulation and are crucial for intraoperative neurophysiological monitoring (IONM) [22,23].

Optical sensing technologies utilize light-based measurement principles to extract physiological information non-invasively [24]. Photoplethysmography (PPG) systems employ light absorption characteristics to monitor cardiovascular parameters including heart rate, blood oxygen saturation, and blood pressure variations [25]. Near-infrared spectroscopy (NIRS) can track changes in oxygenated and deoxygenated hemoglobin concentrations in various tissues, useful for monitoring cerebral oxygenation and muscle metabolism [26,27,28].

Bioimpedance measurement systems analyze the passive electrical properties of biological tissues [29]. Bioelectrical Impedance Analysis (BIA) estimates body composition parameters including fat mass, fat-free mass, and total body water [30,31]. Impedance cardiography (ICG) derives important hemodynamic parameters such as stroke volume, cardiac output, and systemic vascular resistance, providing complementary information to electrical heart monitoring [32].

Thermal sensing technologies include contact-based approaches using thermistors and non-contact infrared thermometers for skin temperature monitoring and thermal mapping [33]. These systems are particularly valuable for assessing autonomic function, detecting inflammatory conditions, immunological disorders, and monitoring wound healing processes [34].

Biochemical sensing represents the emerging frontier, encompassing both invasive and non-invasive approaches for continuous biomarker measurement [35,36]. These sensors can detect specific biological molecules such as nucleic acids, enzymes, antibodies, peptides, or proteins with high accuracy, lower production costs, and reduced assay time [36]. Continuous glucose monitoring (CGM) systems utilize subcutaneous enzyme-based sensors for real-time glucose tracking [37,38]. Non-invasive sweat-based sensors measure electrolytes, metabolites, and hormones through various detection principles [39,40].

Wireless Body Area Networks (WBANs) are wearable devices or sensors placed on, in, or around the human body to monitor physiological data as heart rate, pulse rate, respiratory measurement, temperature, blood pressure, and other biometric signals [41,42]. These devices consist of sensor nodes and a gateway node to convert data collected from the body in digital format, enabling wirelessly remote monitoring [42]. WBANs offer numerous applications in healthcare for continuous patient monitoring, disease detection, sports training, and multimedia communication [43].

### 2.2. Environmental Sensing Technologies

Environmental sensing provides the essential contextual framework for accurate interpretation of physiological signals, enabling distinction between internally driven pathological states and environmentally modulated physiological responses (Table 1).

Atmospheric condition monitoring includes precision temperature, humidity and air quality sensors that track thermal environment parameters essential for interpreting thermoregulatory responses [44,45]. Barometric pressure sensors detect altitude changes and weather-related pressure variations that influence cardiovascular function, respiratory physiology, and various pathological conditions [46]. Air quality sensors monitor particulate matter (PM1, PM2.5, PM10), nitrogen oxides, ozone, carbon monoxide, and other pollutants that may trigger physiological stress responses, inflammatory processes, and autonomic nervous system alterations [47]. Advanced multi-gas sensor arrays can identify complex pollution mixtures and their specific physiological impacts [48].

Chemical environment assessment encompasses volatile organic compound (VOC) sensors that detect organic chemicals affecting respiratory health and cognitive function [49,50]. Metal-oxide (MOX) semiconductor sensors provide broad-spectrum VOC detection, while photoionization detectors (PIDs) offer higher sensitivity for specific compounds [51]. Carbon dioxide monitoring provides indoor air quality assessment, while formaldehyde and specific allergen detection systems enable personalized identification of environmental triggers for allergic individuals [52,53].

Radiation and light exposure monitoring includes ultraviolet radiation sensors for sun exposure assessment and visible light sensors for circadian rhythm regulation [54,55]. Advanced multispectral light sensors can characterize the photic environment in terms of its melanopic equivalent daylight illuminance (EDI), which directly impacts circadian entrainment [56,57].

Acoustic environment characterization involves sound pressure level monitoring for noise exposure assessment and its impact on cardiovascular health and stress levels [58,59,60]. Advanced acoustic sensors incorporate frequency spectrum analysis enabling identification of specific noise types and their physiological effects [61]. Emerging applications include monitoring of vocal biomarkers and cough frequency through wearable acoustic sensors [62,63].

### 2.3. Activity Monitoring Technologies

Activity monitoring captures physical load and movement patterns that directly influence physiological responses and provide essential context for biometric interpretation.

Inertial Measurement Units (IMUs) combine accelerometers, gyroscopes, and magnetometers to quantify physical activity, classify movement patterns, assess gait parameters, and detect falls [64,65]. Modern IMU systems employ sophisticated sensor fusion algorithms that integrate data from multiple inertial sensors to improve motion tracking accuracy [66].

Mechanical cardiography includes seismocardiography (SCG), gyrocardiogram (GCG) and ballistocardiography (BCG) systems that capture cardiac mechanical activity and body micro-movements resulting from cardiovascular function [67,68,69]. These technologies provide valuable complementary information to electrical heart monitoring, particularly during sleep or rest [70]. Several devices, as Mocap systems, combining different sensor types are used in the field of rehabilitation, orthopedics and neurology [71].

Location and context sensing includes integrated GPS and location services that correlate physiological responses with specific environments and activities [72]. Altitude sensors provide essential context for hypoxic responses during mountain activities or air travel [73].

## 3. Existing Multi-Sensor Wearable Devices

The wearable technology market has witnessed progressive integration of multiple sensing modalities, though truly comprehensive biometric–environmental–activity convergence remains relatively limited in commercially available devices. This section provides a detailed survey of existing platforms and their sensing capabilities across the three domains (Table 2).

Commercial fitness and wellness trackers from leading manufacturers typically include optical heart rate monitoring, accelerometry, and increasingly single-lead ECG and SpO_2_ monitoring capabilities [74,75,76]. However, direct environmental sensing remains notably limited, with most devices lacking onboard environmental sensors and instead inferring limited context through location services and connected smartphone data.

Medical-grade wearable monitors such as the Philips Biosensor BX100, VitalConnect VitalPatch, Corsano Cardiowatch 287-2, and Masimo Radius VSM focus on clinical biometric monitoring for specific medical applications [77,78]. These devices typically incorporate medical-grade ECG, high-resolution accelerometry, and respiratory rate monitoring derived from impedance or multi-sensor fusion approaches [79]. While offering robust physiological monitoring capabilities validated for clinical use, they generally lack integrated environmental sensing.

Research and development platforms, including the Empatica EmbracePlus and Shimmer3R GSR+, provide research-grade biometric monitoring capabilities [80,81,82]. These platforms typically include high-quality EDA measurement, PPG-based heart rate monitoring, accelerometry, and skin temperature sensing [83]. While offering superior signal quality and flexibility well suited for research applications, they similarly lack integrated environmental sensing.

Specialized environmental health monitors such as the Atmo Atmotube PRO and Plume Labs Flow focus primarily on environmental sensing within portable form factors [84,85]. These devices typically monitor key air quality parameters including VOCs, particulate matter, temperature, and humidity [86]. However, they generally lack integrated biometric sensing, preventing direct correlation of environmental exposures with physiological responses.

Emerging convergent platforms represent the vanguard of next-generation wearables that actively integrate both biometric, environmental and activity sensing within unified devices [87,88]. These include advanced research prototypes and limited-production devices that demonstrate the practical feasibility and scientific value of true convergent sensing.

### An Exemplary Implementation of Convergent Sensing

In the following paragraphs, we show a prototype (not commercially available) with STMicroelectronics sensors as an exemplary implementation of convergent sensing philosophy, demonstrating the practical utility and technical feasibility of integrated biometric, environmental, and activity monitoring within a unified wearable platform.

System architecture and sensing capabilities:

The prototype integrates a comprehensive sensor array architected for synergistic multi-modal data acquisition. The biometric sensing suite includes medical-grade single-lead ECG for electrical heart activity monitoring, high-resolution EDA/GSR sensors for sympathetic nervous system assessment, bioelectrical impedance analysis (BIA), infrared-based non-contact skin temperature measurement, and a 9-axis IMU. The environmental monitoring suite incorporates precision temperature and humidity sensors, a metal-oxide (MOX) semiconductor-based VOC sensor, a high-resolution barometric pressure sensor, and an ambient light sensor.

Multi-sensing utility demonstration:

The system demonstrates several compelling advantages of convergent sensing:

- Activity monitoring combined with machine learning enables precise physical activity classification and accurate energy expenditure estimation.
- Environmental sensors facilitate real-time thermal comfort assessment using the Predicted Mean Vote (PMV) model, significantly improving the interpretation of skin temperature variations and thermoregulatory responses.
- VOC sensors provide continuous air quality assessment, enabling robust correlation between environmental exposures and physiological stress responses.
- Integrated biometric sensors (ECG, EDA and BIA) offer comprehensive physiological profiling when contextualized with environmental and activity data.

Current limitations: as a research prototype, the device faces several challenges including power management for continuous multi-modal operation, ongoing clinical validation of composite health indices, and the need for further miniaturization for practical wearable implementation. These limitations are typical of early-stage convergent sensing platforms and represent active areas of research and development.

**Figure 2:**
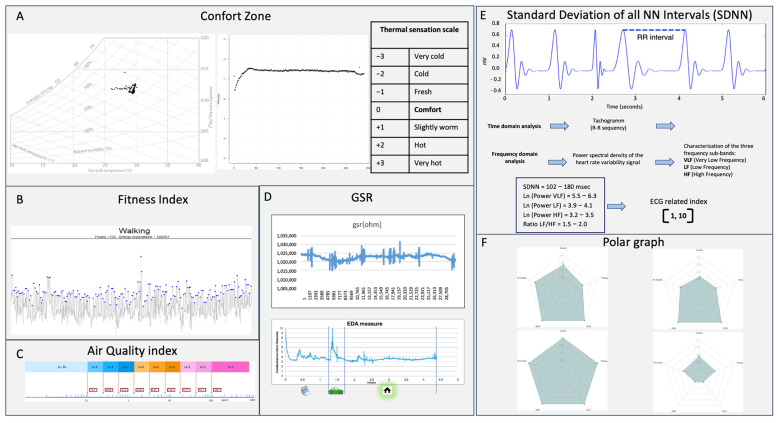
Multi-sensor data fusion for personal well-being assessment. (**A**) Psychrometric Chart and Predicted Mean Vote (PMV) Index; assessment of subjective thermal sensation. (**B**) Fitness Index: objective measure of the person’s caloric expenditure, crucial for metabolic health and energy balance assessment. (**C**) Air Quality Index: a key indicator of respiratory health and overall comfort. (**D**) Galvanic Skin Response (GSR) assessment evaluates emotional stress, anxiety, and cognitive involvement. (**E**) Standard Deviation of all NN Intervals (SDNN); reflects the total variability and adaptability of the Autonomic Nervous System, correlated with physiological stress and cardiovascular health. (**F**) Sensors Radar for health index: a synthetic visual representation (a “radar” or “web” chart) combining values of all indices (**A**–**F**). The resulting shapes represent unique signatures of specific conditions or events (e.g., acute stress, fatigue, optimal comfort state). These signatures can be exploited using ML/AI for automatic diagnosis or prediction of well-being states.

Advanced data fusion and visualization:

Data fusion algorithms can integrate the multiple sensor streams into composite health indices, presented through an intuitive polar graph visualization (Figure 2). This visualization displays five primary indices simultaneously: (A) Comfort Zone Index derived from environmental temperature and humidity sensors using the PMV model; (B) Fitness and Activity Assessment combining motion sensor data with physiological responses; (C) Air Quality Impact integrating VOC measurements with physiological stress markers; (D) Comprehensive Stress Evaluation combining EDA, HRV, and contextual factors; (E) Heart rate variability quantified by calculating the standard deviation of all normal-to-normal (NN) intervals (SDNN) between successive heartbeats. This integrated approach enables intuitive comprehension of complex interrelationships between physiological states, environmental conditions, and activity levels.

(A) Comfort zone index: Thermal sensation assessment derived from environmental temperature and humidity sensors using the Predicted Mean Vote (PMV) model, displayed on an intuitive scale from −3 (Very Cold) to +3 (Very Hot) with optimal comfort at zero [5]. The visualization incorporates color coding and trend indicators to show temporal patterns in thermal comfort.

(B) Fitness and activity assessment: Composite metric integrating motion sensor data for precise activity classification and intensity assessment, combined with physiological responses from ECG and BIA. The display shows current activity type, intensity level, and efficiency metrics based on physiological cost.

(C) Air quality impact: VOC sensor-derived air quality index combined with physiological response indicators, providing crucial context for respiratory and systemic stress. The visualization includes exposure duration weighting and individual sensitivity factors based on historical response patterns.

(D) Comprehensive stress evaluation: Multi-parameter stress assessment combining EDA signals, HRV derived from ECG, and contextual factors from environmental and activity sensors. The display distinguishes between different stress types (physical, psychological, environmental) through visual patterns and provides magnitude indicators.

(E) Heart rate variability: Standard Deviation of all NN Intervals (SDNN) reflects how much the intervals between consecutive heartbeats deviate around the average interval, indicating the overall HRV. The time-domain measure of HRV reflects the total variability and adaptability of the Autonomic Nervous System, correlated with physiological stress and cardiovascular health.

(F) Polar graph: Represents a significant innovation in wearable data visualization, enabling intuitive comprehension of the complex interrelationships between physiological states and environmental conditions. This approach provides a unified health status overview that accounts for both internal and external factors, addressing a critical limitation in conventional wearable interfaces that present isolated metrics without context.

## 4. Discussion

The convergence of biometric, environmental and activity sensing represents a fundamental architectural advancement in wearable health technology, directly addressing critical limitations that have constrained traditional single-domain monitoring approaches [89]. The integrated framework enables genuinely context-aware health assessment that properly accounts for the complex interactions between human physiology, environmental exposures and activity levels [90].

Technical implementation challenges remain substantial and include sensor miniaturization and electromagnetic coexistence, sophisticated power management strategies, precise temporal synchronization across heterogeneous sensor streams, and substantial computational requirements for real-time multi-sensor data fusion [91,92]. These challenges demand sophisticated engineering solutions including adaptive sampling algorithms, hierarchical sensor architectures that minimize interference, and hybrid processing approaches [93].

Clinical Validation Considerations must address the novel challenges posed by composite health indices derived from multiple sensor streams [94]. Traditional medical device validation approaches focused on individual parameter accuracy are insufficient for systems that generate integrated assessments through complex data fusion algorithms [95,96]. New validation frameworks must assess the clinical utility and decision-making impact of these integrated outputs [97,98].

Data Interpretation and Visualization represent a critical challenge in making complex multi-modal data accessible, actionable, and clinically meaningful [99,100]. Conventional approaches to wearable data presentation are inadequate for representing the rich contextual relationships in multi-modal data. Advanced visualization strategies, such as the polar graph approach, play a crucial role in communicating integrated health status while preserving the contextual relationships.

Machine Learning and AI Integration are essential for advanced data fusion and interpretation in convergent sensing platforms [101,102]. ML algorithms can identify complex patterns across multiple data streams, enabling personalized baseline establishment, anomaly detection, and predictive analytics. However, these approaches require large, annotated datasets and careful validation to ensure clinical reliability.

Privacy and ethical considerations assume heightened importance with convergent sensing due to the unprecedented intimacy and comprehensiveness of the collected data. The combination of detailed physiological information with precise environmental, location and activity context creates exceptionally sensitive datasets. Transparent data governance policies and privacy-preserving computation techniques are crucial for maintaining user trust [103].

Future directions for convergent sensing platforms include several prioritized research pathways.

1. Development of standardized validation frameworks for multi-modal health indices.
2. Advancement in ultra-low-power sensor technology and energy-efficient communication protocols.
3. Creation of large, annotated multi-modal datasets for ML algorithm training.
4. Implementation of closed-loop systems that deliver personalized interventions based on integrated monitoring.
5. Establishment of ethical guidelines and regulatory pathways for convergent devices.

As these technologies mature, they have the potential to transform healthcare from a reactive model focused on disease treatment to a proactive paradigm centered on health optimization and preservation.

## 5. Conclusions

The integration of biometric, environmental, and activity sensing in wearable platforms marks a significant evolutionary advancement in personal health monitoring, enabling a comprehensive assessment that properly accounts for physiological status within relevant contextual frameworks [104]. This convergent approach directly addresses fundamental limitations of traditional monitoring systems by providing essential contextual layers necessary for accurate interpretation of physiological signals.

The STMicroelectronics prototype exemplifies the practical implementation of this convergent sensing paradigm, demonstrating how integrated data from sensor domains can be synthesized into actionable health insights through sophisticated data fusion and intuitive visualization. While substantial technical and clinical challenges remain, particularly regarding validation, power management, and data interpretation, the potential benefits of convergent sensing for personalized health management are profound.

The continued convergence of sensing technologies, advanced data analytics, machine learning and user-centered design will drive the development of increasingly sophisticated health monitoring platforms that account for the complex interplay between individual physiology, environmental exposures and activity patterns [105,106]. This technological evolution, coupled with appropriate attention to validation, privacy, and usability considerations, will ultimately support more effective, personalized, and preventive health management strategies across diverse populations and healthcare scenarios [107]. The vision of truly holistic health monitoring that seamlessly integrates internal physiological status with external environmental and activity context represents a compelling future direction for wearable technology with transformative potential for both individual health and public health surveillance [108].

## Figures and Tables

**Table 1:** Environmental context for biometric interpretation: summary of the interrelationships between environmental factors, relevant biometric parameters, and their clinical significance, illustrating how contextual sensing enables more accurate health assessment.

Environmental Factor	Sensing Technology	Relevant Biometric Parameters	Clinical Significance
Thermal Environment	Temperature/ Humidity Sensors	Skin temperature, Heart rate, EDA, Peripheral blood flow	Distinguishes thermoregulatory stress from pathological tachycardia; identifies heat/cold stress conditions
Air Quality	VOC, PM2.5, NO_2_, O_3_ Sensors	Respiratory rate, HRV, SpO_2_, Cough frequency, Inflammatory markers	Identifies environmental triggers for asthma/COPD exacerbations; links pollution exposure to cardiovascular events
Atmospheric Pressure	Barometric Pressure Sensors	Heart rate, Blood pressure, Cerebral blood flow, Headache occurrence	Correlates pressure changes with migraine attacks, joint pain, and cardiovascular symptoms
Light Exposure	UV/VIS Light Sensors	Sleep quality, Melatonin rhythm, Activity patterns, Cognitive performance	Links circadian disruption to metabolic syndrome, cardiovascular risk, and mood disorders
Noise Pollution	Sound Pressure Sensors	HRV, Blood pressure, Stress hormones, Sleep architecture	Quantifies the cardiovascular impact of environmental noise; identifies noise-induced sleep disruption
Altitude/ Hypoxia	Barometric Pressure, GPS	SpO_2_, Heart rate, Respiratory rate, Exercise capacity	Monitors acclimatization status; detects early signs of altitude sickness
Chemical Exposures	Specific Gas Sensors	Respiratory function, Inflammatory markers, Liver enzymes	Identifies occupational and environmental chemical exposures; monitors individual susceptibility

**Table 2:** Multi-sensor wearable devices comparison. Comparative analysis of multi-sensor wearable devices across different categories, highlighting their sensing capabilities, applications, and limitations.

Device Category	Example Devices	Biometric Sensors	Environmental Sensors	Key Applications	Limitations
Consumer Fitness	Apple Watch Series, Fitbit Sense, Garmin Venu	ECG, PPG, HRV, Accelerometer, Temperature, Respiration Rate, SpO_2_	Indirect only (via smartphone or inference)	Fitness tracking, wellness monitoring, activity and sleep assessment	Not medical-grade; physiological and contextual data often inferred rather than directly measured
Clinical Monitoring	VitalConnect VitalPatch, Corsano CardioWatch 287-2, Masimo Radius VSM, Philips Biosensor BX100	ECG, Accelerometer, Respiration Rate, Temperature, SpO_2_ (device-dependent)	None	Remote patient monitoring, clinical trials, hospital and step-down surveillance	No direct environmental context; typically confined to clinical or regulated settings
Research Platforms	Empatica EmbracePlus, Shimmer3R GSR+	EDA, PPG, Accelerometer, Temperature, optional EEG/EMG modules	None	Psychophysiology research, stress and affective computing, sleep and behavior studies	Environmental exposure must be measured separately; not designed for routine clinical deployment
Environmental Focus	Atmo Atmotube PRO, Plume Labs Flow	None	VOC, PM1/2.5/10, NO_2_, CO_2_, Temperature, Humidity, Pressure	Personal air quality monitoring, exposure and pollution assessment	No physiological monitoring; limited insight into health impact without biosignals
Convergent Prototypes	STMicroelectronics Platform, Research prototypes	ECG, EDA, PPG, BIA, Temperature, Accelerometer	VOC, Temperature, Humidity, Pressure, Light	Comprehensive health-environment interaction studies	Limited availability; early development stage; validation ongoing

## Data Availability

No new data were created or analyzed in this study.

## Abbreviations

The following abbreviations are used in this manuscript:

BAEP	Brainstem auditory evoked potential
BCG	Ballistocardiography
BIA	Bioelectrical impedance analysis
COPD	Chronic Obstructive Pulmonary Disease
ECG	Electrocardiogram
EDA	Electrodermal activity
EMG	Electromyography
EEG	Electroencephalography
GCG	Gyrocardiogram
GSR	Galvanic Skin Response
IMU	Inertial Measurement Unit
MEP	Motor evoked potential
NIRS	Near-infrared spectroscopy
HRV	Heart Rate Variability
PIDs	Photoionization detectors
PMV	Predicted Mean Vote
PPG	Photoplethysmography
SCG	Seismocardiography
SDNN	Standard Deviation of all NN Intervals
SSEP	Somatosensory evoked potential
VEP	Visual evoked potential
VOC	Volatile Organic Compound
WBANs	Wireless Body Area Networks
